# Chlorosulfonic Acid Stretched Carbon Nanotube Sheet for Flexible and Low-Voltage Heating Applications

**DOI:** 10.3390/nano11082132

**Published:** 2021-08-21

**Authors:** Daniel Rui Chen, Megha Chitranshi, Paa Kwasi Adusei, Mark Schulz, Vesselin Shanov, Marc M. Cahay

**Affiliations:** 1 Department of Mechanical and Materials Engineering, University of Cincinnati, Cincinnati, OH 45221, USA; aduseipi@mail.uc.edu (P.K.A.); shanovvn@ucmail.uc.edu (V.S.); 2 Department of Electrical Engineering and Computer Science, University of Cincinnati, Cincinnati, OH 45221, USA; cahaymm@ucmail.uc.edu; 3 Department of Chemical and Environmental Engineering, University of Cincinnati, Cincinnati, OH 45221, USA

**Keywords:** CSA-stretching, CNT, FC-CVD

## Abstract

The carbon nanotube (CNT) is celebrated for its electrothermal property, which indicates the capability of a material to transform electrical energy into heat due to the Joule effect. The CNT nanostructure itself, as a one-dimensional material, limits the electron conduction path, thereby creating a unique heating phenomenon. In this work, we explore the possible correlation between CNT alignment in sheets and heating performance. The alignment of carbon nanotubes is induced by immersion and stretching in chlorosulfonic acid (CSA) solution. The developed CSA-stretched CNT sheet demonstrated excellent heating performance with a fast response rate of 6.5 °C/s and reached 180 °C in less than 30 s under a low voltage of 2.5 V. The heating profile of the stretched CNT sheet remained stable after bending and twisting movements, making it a suitable heating material for wearable devices, heatable smart windows, and in de-icing or defogging applications. The specific strength and specific conductance of the CSA-stretched CNT sheet also increased five- and two-fold, respectively, in comparison to the pristine CNT sheet.

## 1. Introduction

Since the discovery of carbon nanotubes (CNTs) through Iijima’s famous research in 1991 [1], there has been an explosion of interest in the search for new CNT applications, mainly due to its superior properties—such as electrical [2,3,4] and thermal conductivity [5,6]—as well as its ability to provide desirable mechanical support [7,8]. The theoretical current density of CNT is three times higher than copper [4] and its thermal conductivity of 3500 W/(m K) is the highest of all known materials [6], while it also retains superior mechanical flexibility and stability [9], therefore making CNT a great candidate for high-performance flexible electronics.

In addition to the many CNT-based flexible electronics that have been developed—including strain sensors [10,11], electronic skins (E-Skins) [12], and high-performance flexible integrated circuits (ICs) [13]—there is growing interest in the application of CNTs as flexible macroscale heaters, with lower power-consumption, light weight, and fast heat response. Such heating devices holds great promise in solving many real-world problems—notably de-icing applications on airplanes in the aerospace industry and heating apparel for patients with health problems such as hypothermia, frostbite, flu, etc., in the healthcare system [14].

The majority of heaters are based on the electrical-thermal effect, which is the phenomenon that enables materials to convert electrical energy to thermal energy; the heat by-product is mainly generated from the inelastic collision between phonons and electrons in an electric field [15]. Currently, materials based on high-resistance elements such as Ni-Cr (nichrome) or Fe-Cr-Al (Kanthal) are primarily used in heat-generating appliances. There are, however, many constraints associated with these materials, such as their rigidity and their intolerance towards acids and bases—limiting their broader application [16]. CNTs, on the other hand, are free from such restrictions. A detailed review discussed their wet chemistry and solid phase transfer production method for electrothermal applications: the first being a multi-step process requiring harsh chemicals, and the latter being a single-step process not requiring any pretreatment steps that harm the properties of the material [17].

In this work, the floating catalyst chemical vapor deposition (FC-CVD) method was used to manufacture CNT. FC-CVD is well known for its continuous process and high-purity output [18,19,20,21]. It offers a cost-effective way to manufacture CNT and, more significantly, it provides a final stage of development from assembled nanomaterials into macroscopic scaffolds which can be in sheet or thread form. In addition, chlorosulfonic acid (CSA)-assisted stretching is used in tandem as a post-processing step to align the CNT assembly and further improve its properties. We show that CSA-stretched CNT has three times the density, twice the specific strength, and five times the specific conductance of the as-synthesized CNT. The CSA-stretched CNT with the best properties was able to heat up to 180 °C, when a small voltage of 2.5 V was applied, in less than 30 s—showing great promise as a heating element in small-voltage flexible heaters.

## 2. Experimental Section

### 2.1. CNT Sheet Fabrication

CNT sheet was synthesized using the floating catalyst chemical vapor deposition (FC-CVD) method. In this method, feedstock—which consists of a carbon precursor, catalyst, and sulfur as a promoter—was injected into a tube in a high-temperature furnace. The CNT sock was collected on the other end of the tube on a rotating drum to form a sheet (Video S1). The temperature of the furnace in the heating zone was approximately 1400 °C. The fuel used in the synthesis is a mixture of ferrocene (Sigma-Aldrich, Inc., St. Louis, MO, USA) methanol (Thermo Fisher Scientific, Waltham, MA, USA), and thiophene (Sigma-Aldrich, Inc., St. Louis, MO, USA). The fuel was injected into the reactor using a syringe pump which is connected to the atomizer and then to a mixer where it mixes with the carrier gases. The carrier gas flow can vary depending on the synthesis process. Usually, the H_2_ gas flow is 100 sccm and the Ar gas flow varies from 1000 to 2000 sccm. Various factors—such as the synthesis temperature, gas flow rate, and the pressure inside the glove box—can affect the condition of the sock; hence, the process must be carefully tuned to avoid breaking of the sock. Further information on our synthesis process can be found in our previous works [22,23].

### 2.2. CSA-Assisted Stretching

**Figure 1:**
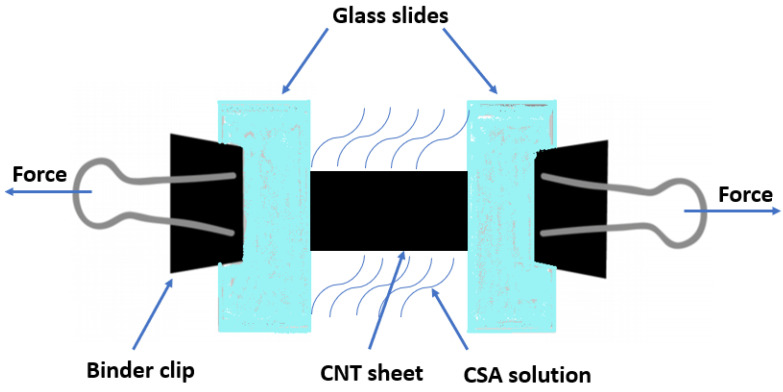
Schematic drawing of the stretching process.

The as-synthesized CNT sheet was cut into a rectangular strip with the longer side as the winding direction (length 20 mm × width 2 mm × thickness 30 μm) (Figure 1). The shorter sides of the strip were clamped between two glass slides at both ends (Video S2). The whole setup was transferred into a petri dish and immersed in CSA (Sigma-Aldrich, Inc., St. Louis, MO, USA) solution. After 1 min of immersion, small and incremental forces were applied from both sides to ensure uniform stretching. The stretched samples were then immersed in chloroform (Sigma-Aldrich, Inc., St. Louis, MO, USA) for 24 h to wash off the residual CSA and coagulate CNTs.

### 2.3. Material Characterization

The orientation and the surface morphology of the carbon nanotube sheet were characterized by scanning electron microscopy (SEM) (FEI SCIOS dual beam, 5 kV, Waltham, MA, USA). For SEM characterization, no additional conducting coating was needed due to the high electrical conductivity of the CNT samples. Raman spectroscopy (Reinshaw inVia, Wharton Andech, UK) via a 514 nm Ar-ion laser with a laser spot size of ~1 μm^2^ was used for structural characterization and to assess the quality of the CNT sheet. Tensile testing was performed using an Instron 5948 machine. A laser micromachining system (Oxford Laser A-Series, Didcot, UK) was used to cut the CNT sheet samples. The electrical conductivity of the sheet was measured using a four-probe technique (Jandel RM3000, Leighton Buzzard, UK).

### 2.4. Heating Performance Measurement

A heating performance test of the CNT with different stretching ratios was carried out on a custom-designed sample testing setup with glass slide as sample substrate and copper tape as electrodes on each side. Silver conductive paint (TED PELLA, INC., Redding, CA, USA) was used in between the CNT and copper tape surface to ensure minimal contact resistance. The temperature input device (National Instruments, Austin, TX, USA)—a thermocouple measurement device—was used to take temperature measurements of the device while simultaneously logging data into the PC. Other custom-made electronics included a voltage stabilizer (XY-SK35 CNC buck boost, Econede) and a time relay device (XY-WJ01 Delay Relay Module, Hardware), which were used to ensure constant voltage supply and more accurate control of heating and cooling time, respectively.

## 3. Results and Discussion

### 3.1. The Effect of CSA-Stretching

For this research, we used the CSA-assisted stretching approach to improve the strength and conductivity of CNT sheet. CNT dissolves in CSA via reversible protonation [24]. Dissolution and dispersion occur when the delocalized positive charge induces a repulsive force between CNTs to counteract the van der Waals forces. The CNT alignment is further enhanced by the stretching process described below [24,25,26].

The degree of stretchability in CSA solution correlates directly to the mechanical properties, as well as the graphitization of the original CNT materials [24,26]. The versatility of the FC-CVD method enables the production of CNT with a relatively high degree of graphitization under a hydrogen environment. The CNT sample used in this paper had an average I_G_/I_D_ of 8 (Figure S1A), speaking to its high degree of graphitization. I_G_ is known as the peak intensity of the G-band (1582 cm^−1^), it comes from the carbon–carbon bonds stretching within the graphene plane and correlates to the graphitized CNT structure [27] while I_D_ refers to the peak intensity from the D-band (1348 cm^−1^), which is the result of the double-resonance Raman effect in sp^2^ carbon and corresponds to the defective CNT structure (amorphous or disordered carbon).

Due to the high graphitization of as-synthesized CNT, we achieved double the length via stretching (100% stretching ratio) without breaking. This process was then ended by immersion in chloroform for 24 h. The chloroform was used to wash off the residual CSA and mitigate the complex influences of doping chemical species, as well as to coagulate and form densely packed structure, an acetone wash was further used to clean out the CSA and densify the materials [26]. Although most of the CSA protonation was eliminated during this process, a small remnant of the CSA still contributed to the chemical doping and enhanced the conductivity of the sheet without affecting the general property of the materials.

### 3.2. The Improvement of Mechanical and Electrical Properties

The CSA-assisted stretching consisted of two steps: the first step being the CSA immersion, and the second being the gradual stretching after immersion. The immersion time was 1 min long as it was found that immersion for less than 1 min did not provide sufficient time to cause structural rearrangement, while excessive immersion causes complete dissolution and can disassemble the well-defined CNT sheet. Hence, 1 min immersion time was utilized to provide the maximum possible stretchability.

**Figure 2:**
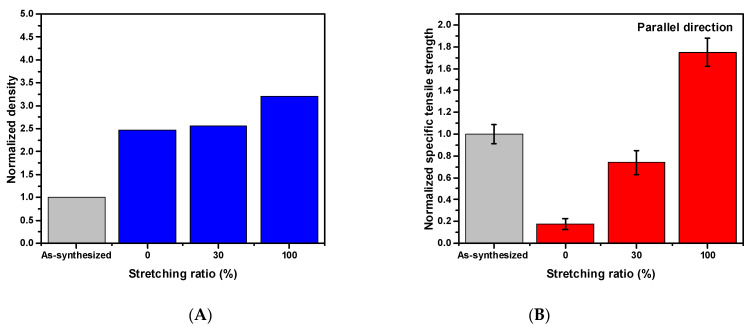

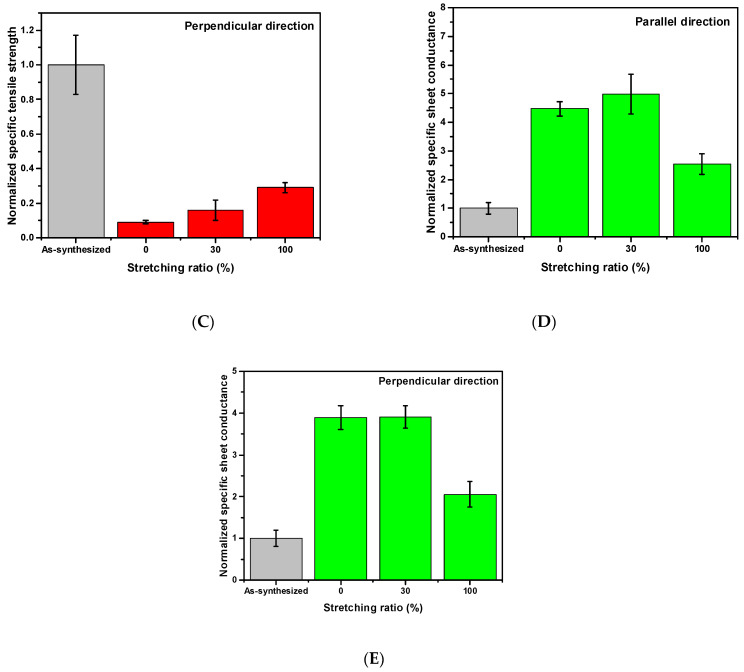
Evolutions in (**A**) normalized density, (**B**) normalized specific tensile strength in parallel direction, (**C**) normalized specific tensile strength in perpendicular direction, (**D**) normalized specific sheet conductance in parallel direction, and (**E**) normalized specific sheet conductance in perpendicular direction. During the CSA densification and stretching process, parallel direction refers to the winding direction from the synthesis process, whereas perpendicular direction indicates the direction perpendicular to winding direction. All values are normalized to those of as-synthesized CNT.

The evolution of mechanical and electrical properties during densification and stretching is examined in this section. For the sake of clarity, as-synthesized CNT was used as the reference sample, and samples with different stretching ratios were normalized to the reference one. The density of the CNT sheet was found to increase by a multiple of 2.5 when immersed in CSA (0% stretching ratio) and increased 3-fold when the immersion was coupled with stretching by 100%, as shown in Figure 2A. The density increase in CNT was largely due to CSA’s ability to penetrate the CNT sheet and solvate CNT by protonation [28].

As the stretching ratio increased from 0% to 100%, the normalized specific tensile strength increased linearly from 0.2 times to 1.7 times that of the as-synthesized CNT in the parallel direction, i.e., along the winding direction (Figure 2B). Solely CSA immersion did not introduce any changes to the mechanical properties of the material, the increased density, however, further reduced the specific tensile strength. The density from 0% stretching to 100% stretching shows a similar increment of 2.5, 3.0 times respectively. Thus, the vast improvement in normalized tensile strength in the parallel direction is attributed to CNT alignment from stretching (since the stretching was in the parallel direction). Increased CNT alignment enabled more uniform load distribution along the tube direction, which is the strongest direction, and collectively enhances the tensile strength of the CNT sheet. In contrast, normalized tensile strength in the perpendicular direction (Figure 2C) degraded compared with that of the as-synthesized CNT because of the weak van der Waals force. There are fewer CNTs in that direction to bear the load, whereas the as-synthesized CNT, by virtue of the misalignment, has more CNTs that can bear the load. The value of specific stress for each sample can be found in Figure S2.

The sheet conductance in both the parallel and perpendicular direction were measured and are presented in Figure 2D,E. The 30% stretching ratio resulted in a 5-fold increase in specific sheet conductance compared with the as-synthesized CNT in the parallel direction. The 30% stretching ratio gave the highest sheet conductance values amongst all three samples with different stretching ratios. The sheet conductance of the as-synthesized CNT in parallel and perpendicular directions can be found in Figure S3. The optimization of sheet conductance can be understood as the optimal stretching ratio needed to form the conductive CNT network. It is possible that the 0% and 100% stretching ratios were not able to provide the optimal conductive pathway due to inadequate and excessive stretching, respectively. Protonation could also be a major factor of the conductivity increase in the 30% stretched sheet due to the residual CSA on the sheets.

Joule’s Law, P = V^2^/R (where P is the electrical power, V is the input voltage, and R is the resistance), is used to calculate the conversion of electrical energy to thermal energy with time. Therefore, a decrease in resistance reduced the voltage needed to reach the steady-state temperature for constant power output. After the CSA-stretching process, protonation and better interconnectivity along the stretching direction enable the 30% stretched CNT sample to have the lowest specific resistance value, which is ideal for use as a low-voltage heater.

### 3.3. The Analysis of Alignment

**Figure 3:**
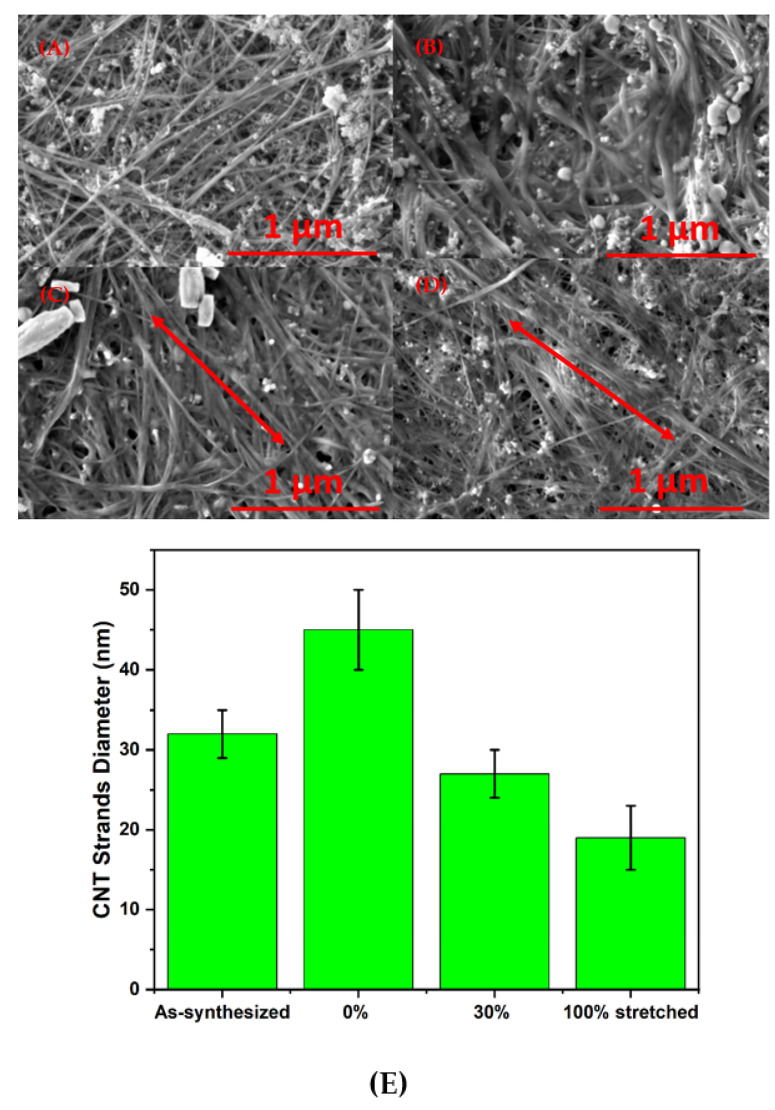
SEM images of as-synthesized (**A**), 0% stretched (**B**), 30% stretched (**C**), and 100% stretched (**D**) CNTs. The stretching direction is indicated by the red arrows in (**C**,**D**). The CNT strands diameter distribution from differently stretched CNTs is shown in (**E**).

The quantifiable alignment was measured through the strand diameter distribution (Figure 3E). The CNT strand consists of a bundle of individual CNTs combined with each other under weak Van der Walls forces, after immersion in CSA solution for 1 min, due to the penetration of the CSA solution, the strand is swollen and its diameter is increased, as seen in Figure 3B. After the immersed CNT was stretched by 30% and 100% (Figure 3C,D), the diameter of the CNT strand gradually decreased until, eventually, the strand diameter of CNT strand fell below that of the as-synthesized CNT (Figure 3A), which suggests increased alignment and a more closed, packed structure. The morphology changes contributed significant to the overall densification and mechanical enhancement of the sheet. Singular directional stretching also led to the increased anisotropy of the materials, as is evident from the mechanical measurement (Figure 2B,C). The two directions (parallel and perpendicular) demonstrated a discrepancy, as the parallel direction showed better tensile strength than the perpendicular direction did. After the stretching and coagulation, CNTs have a larger contact surface area with each other. Besides the improvement in the mechanical properties of the sheet, conductivity also increased 4-fold due to the CSA doping effect.

### 3.4. Heating Performance of CSA-Stretched CNTs

To investigate the electrothermal properties of various CSA-stretched CNT sheets, a series of heating experiments were conducted. Each voltage was applied at 0.5 V intervals for 5 min and the performance was recorded. It was noted that the temperature of the electrothermal sheet decreased to room temperature when the power was turned off. A comparative analysis of heating properties based on carbon nanotube sheets is shown in Table 1. The CSA-stretched CNT sheet showed excellent heating performance with fast response times, as compared with the other CNT-based heaters. Additionally, CSA-stretched CNT heaters do not require any substrate, unlike other carbon nanotube-based heaters. The other CNT-based heaters required extremely high voltage and time to attain peak temperatures (below 100 °C).

**Figure 4:**
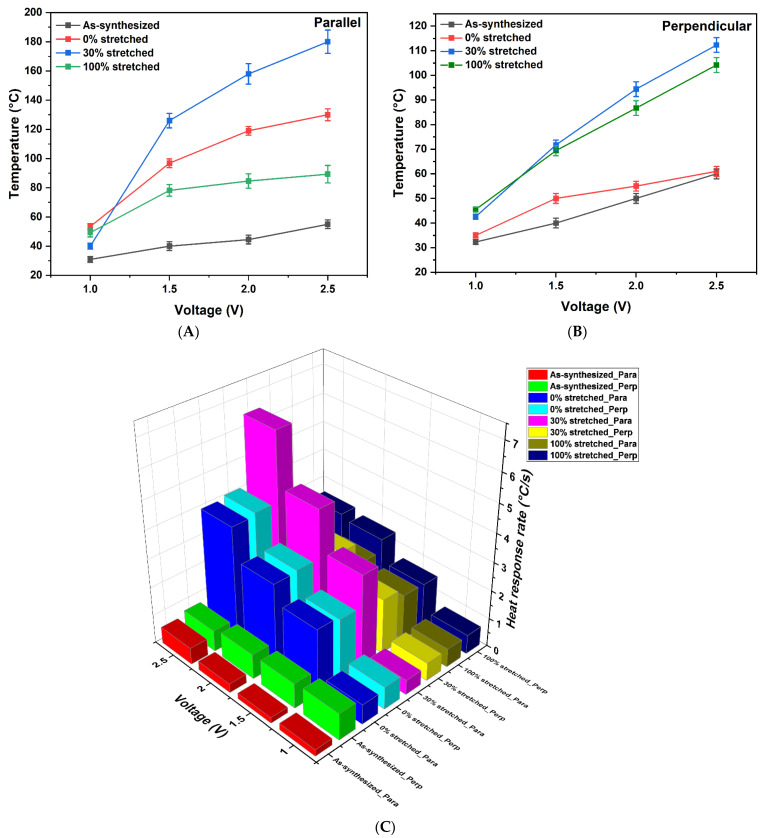
Heating performance of as-synthesized and CSA-stretched CNT samples from 1 V to 2.5 V in parallel direction(**A**) and perpendicular direction (**B**)**,** as well as the heating response rate of these samples in both directions (**C**).

Figure 4A,B show the temperature profile of the CNT samples at different DC voltages. Pristine samples, along with 0%, 30%, and 100% CSA-stretched samples, were tested in both parallel and perpendicular directions. The voltage was increased from 1 V to 2.5 V in 0.5 V increments over 5 min intervals. In Figure 4A, the pristine sample did not show much increase in temperature and the steady state temperature for this sample was approximately 55 °C, with a very slow response rate of ~0.5 °C/s (Figure 4C). The sample with 0% stretching showed good heating response and reached 130 °C within a few seconds at 2.5 V with 4 °C/s. The response time of the sample increased with the increase in voltage which follows the Joules heating law. The electrical heating power is proportional to the square of the applied voltage; hence, the higher the applied voltage, the higher the heating rate. The sample with 30% stretching showed an excellent heating rate, starting at 40 °C at 1 V and reaching the maximum temperature of 180 °C at 2.5 V, with response times of 0.5 °C/s and 6.5 °C/s at 1 V and 2.5 V, respectively. After CSA stretching, CNT electrothermal sheets had smaller diameter and denser CNT strands, which resulted in a less electrically resistive pathway. The high electrical conductivity was attributed to a high packing density and fewer impurities, which avoided the electron scattering responsible for suppressing electron transport [35]. The heating performance of the sample with 100% stretching was lower than that of the 0% and 30% stretched samples. The lower performance is attributed to its lower specific sheet conductance, as compared with other samples. This may be due to exceeding the optimum stretching ratio, after which the CNTs dissemble. Another reason may be that the conductivity cannot be improved above a certain point by structural rearrangement [28]. The steady state temperature profiles for all samples are shown in Figure S4.

The heating performance in perpendicular direction is shown in Figure 4B. The heating performance of the samples followed a similar trend as in the parallel samples, except the sample with 0% stretching showed lower performance compared with the rest of the samples, which may be due to the swelling effect cause the “parallel separation” of CNTs in perpendicular direction. The reason for this lower performance in perpendicular direction is that heat is conducted in the direction of the nanotubes, the stretching action forces the CNTs to be aligned in the parallel direction. Hence, the heat is readily transported in parallel direction instead of perpendicular direction [36]. The sample with 30% stretching demonstrated better performance in the perpendicular direction as compared with the other samples and reached a maximum temperature of 112.3 °C at 2.5 V with a heat response rate of 2.9 °C/s. The detailed heating information of various samples can be found in Figure S4 and Table S1.

Overall, the 30% stretched sample performed better amongst all samples, which gives insight into the optimum stretching ratio. The better performance of the 30% CSA-stretched CNT sheet is the result of the lower sheet resistance and the stretching producing a direct and aligned conductive path between CNTs. The high electrical conductance of the sample in both directions also proves this point (Figure 2).

### 3.5. Heating Performance of 30% Stretched CNT

**Figure 5:**
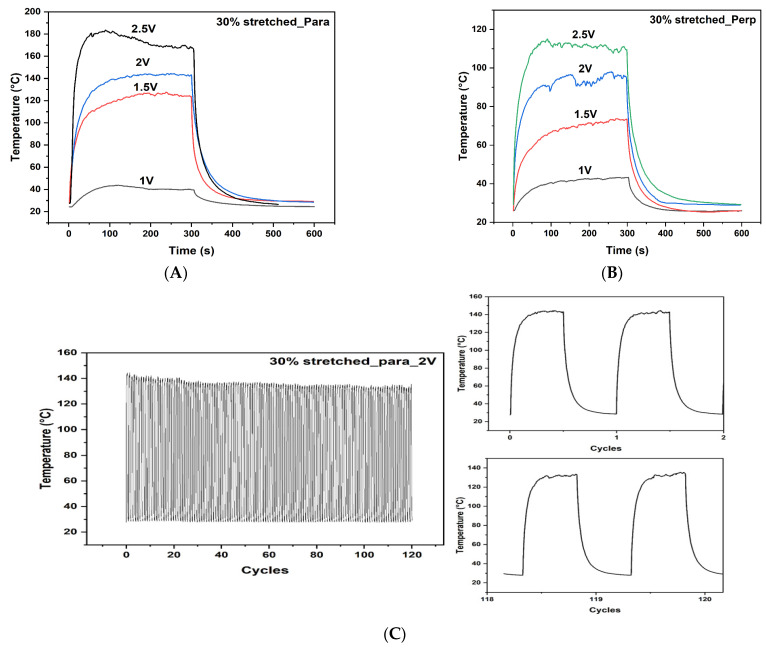

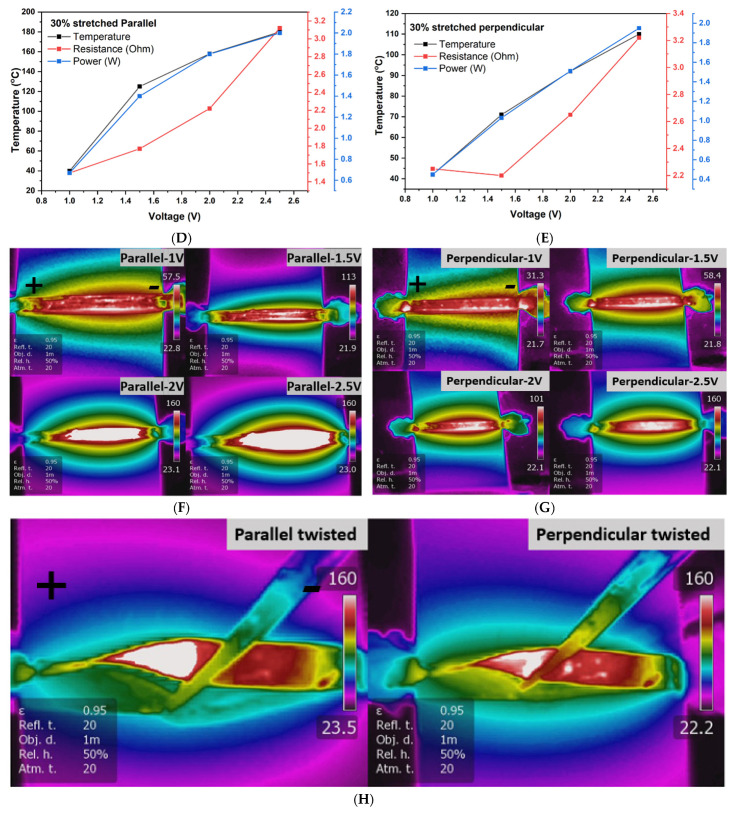
Heating performance of the 30% CSA-stretched CNT in parallel direction (**A**) and perpendicular direction (**B**); heating performance of the 30% stretched parallel sample over 120 cycles with the first and last two cycle performance (**C**); power-voltage-temperature-resistance plot for the 30% CSA-stretched CNT in parallel direction (**D**) and perpendicular direction (**E**); thermal images of the 30% CSA-stretched CNT in parallel direction (**F**), perpendicular direction (**G**), and with twisting movement (**H**).

The heating profile for the 30% CSA-stretched sample, with respect to the applied voltage, is shown in Figure 5A,B. For parallel direction, steady state temperatures for the 30% stretched sample were 40 °C, 125 °C, 158 °C, and 180 °C at 1 V, 1.5 V, 2 V, and 2.5 V, respectively. The exceptional heating performance of this sample at exceptionally low voltage is due to its excellent current-carrying capability. The CSA stretching exfoliated and doped the CNT and resulted in a more conductive sample [37]. The sample with 30% CSA stretching showed a higher heating rate with a high response time and can be used in de-icing and defrosting applications.

The cooling time of the samples was greater than the heating time which can be attributed to the accumulation of heat. Figure 5B shows the heating profile in perpendicular direction. The steady state temperatures for the 30% stretched sample in perpendicular direction were 42 °C, 71 °C, 92 °C, and 110 °C at 1 V, 1.5 V, 2 V, and 2.5 V, respectively. In this direction, the heating performance was not as good as in its parallel direction sample due to the unavailability of a continuous conduction path.

Figure 5C shows the heating performance of the stretched electrothermal sheet over 120 cycles. The heating profile remained with little degradation over the entire time, which demonstrated the great stability and repeatability of the electrothermal sheet over longer periods of operation. A constant voltage of 2 V was applied to the CNT sheet with a heating/cooling interval of 5 min each. The heating profile of the first two and last two cycles are shown in the right side of Figure 5C.

Figure 5D,E show the temperature-voltage-resistance-power relationship in both parallel and perpendicular directions. In parallel direction, the temperature was increased with the increase in voltage, the resistance was also increased from 1.5 ohms to 3.12 ohms, and the power was varied from 0.67 W to 2 W, which indicate the material can be used in low power applications. In perpendicular direction, the sample showed the similar behavior with respect to voltage and temperature, the resistance was increased from 2.25 ohms to 3.2 ohms, and power was varied from 0.45 W to 1.95 W. The increase in resistance is due to the oxidation at higher temperatures.

Heat generation depends upon the input power and surface area of the heating film. When the input power is applied to the CSA-stretched sheet, heat is generated according to the Joule’s law and is a combination of conduction of the substrate, convection of the surrounding air, and radiation from the hot surface of the heater [26,38,39].

Q_in_ = Q _out_ (1)

Q_uota_ = Q _Convection_ + Q _Conduction_ + Q _Radiation_ (2)

The heat loss due to conduction can be neglected as CSA-stretched CNT sheet had no substrate. Hence, the above equation can be written as:

Q _out_ = Q _Convection_ + Q _Radiation_ (3)

The convection of the surrounding air can be expressed as:

Q _convection_ = h _convection_ × A _convection_ × ΔT (4)

where h _convection_ is the convective heat transfer coefficient, A _convection_ is the surface area, and ΔT is the difference between the CNT sheet surface and air.

The radiation heat loss can be expressed as:

Q _Radiation_ = ε × σ × A _Radiation_ × ΔT^4^ (5)

where ε is the surface emissivity, σ is the Stefan–Boltzmann constant (5.67 × 10^−8^ W m^−2^^·^ C^−4^), and A _Radiation_ is surface area of the radiation.

An IR camera was used to monitor the uniformity of heat distribution at each input voltage in both parallel and perpendicular directions. The thermal infrared images showed the excellent heating properties of the sheet (Figure 5F,G). The temperature was distributed uniformly over the CNT sheet. The heating properties of the carbon nanotube sheet remained the same after bending and twisting operations (Figure 5H), which makes it a promising candidate for wearable applications. The electrothermal performance of these carbon nanotube sheets followed Joule’s law.

## 4. Conclusions

In this work, we demonstrated a flexible and low voltage CSA-stretched CNT electrothermal sheet with excellent heating performance. Different stretching ratios were studied, and a comparative analysis of the stretched CNT sheets and as-synthesized CNT sheets revealed an optimum stretching ratio. CNT sheet with 30% CSA stretching reached a steady state temperature of 180 °C within a few seconds, with a 6.5 °C/s response rate, at 2.5 V. It also showed an almost 3-fold increase in density, a 2-fold increase in specific strength, and a 5-fold increase in specific conductance, compared with as-synthesized CNT. The heating performance of the 30% CSA-stretched CNT sheet examined over 120 heating and cooling cycles demonstrated a stable heating behavior. Thermal images confirmed the uniform heating profile after bending and twisting movements of the flexible sheet, making it a promising candidate for wearable applications. The fast-heating response of these flexible sheets at low voltages has great potential for de-icing, defogging, and heating of smart windows, among other applications.

## Figures and Tables

**Table 1:** Heating performance of carbon nanotube-based heaters.

Material	Size (cm × cm)	Voltage (V)	Temperature (°C)	Reference
CSA-stretched CNT	2 × 0.2	2.5	>180	This work
MWCNT on Glass	1 × 1.5	40	80	[29]
MWCNT on PET	0.65 × 0.85	15	77	[30]
SWCNT on PET	4 × 4	12	95	[31]
SWCNT on PET	5 × 5	35	75.5	[32]
CNT/Pd	0.5 × 0.5	5.5	105	[33]
CNT ink on PET substrate	8.5 × 3	35	50 ± 3.8	[34]

## Data Availability

The data presented in this study are available on request from the corresponding author.

## Supplementary Materials

The following are available online at https://www.mdpi.com/article/10.3390/nano11082132/s1, Figure S1: The Raman graph of as-synthesized CNT (A) and as-synthesized CNT after 30% CSA stretched (B), Figure S2: Tensile test of CNT samples after various treatments, Figure S3: Sheet conductance of as-synthesized CNT sheet in parallel and perpendicular direction, Figure S4: Heating performance under different voltages from samples, Table S1: Heating information of various stretched CNT sheets, Video S1: CNT sock collection process, Video S2: CNT CSA-assisted stretching process.
