# Supplementary material for: Chlorosulfonic Acid Stretched Carbon Nanotube Sheet for Flexible and Low-Voltage Heating Applications
Source: Nanomaterials (Basel). 2021 Aug 21;11(8):2132. doi: 10.3390/nano11082132 (PMC8398647; doi:10.3390/nano11082132)
Supplement: Supplementary file 1 [file nanomaterials-11-02132-s001.zip › Supplementary information.pdf]

Supplementary information

Chlorosulfonic Acid Stretched Carbon Nanotube Sheet for Flexible and Low-Voltage Heating Applications

Daniel Rui Chen [1,*], Megha Chitranshi [2,*], Paa Kwasi Adusei [1], Mark Schulz [1], Vesselin Shanov [1,3], Marc M. Cahay [2]

[1] Department of Mechanical and Materials Engineering, University of Cincinnati, Cincinnati, OH 45221, USA

[2] Department of Electrical Engineering and Computer Science, University of Cincinnati, Cincinnati, OH 45212, USA

[3] Department of Chemical and Environmental Engineering, University of Cincinnati, Cincinnati, OH 45221-0012, USA

* These two authors contribute equally for this manuscript

Corresponding Author: Daniel Rui Chen, E-mail: chenru@mail.uc.edu;
Megha Chitranshi, E-mail: chitrama@mail.uc.edu
Mark Schulz, E-mail: schulzmk@ucmail.uc.edu,

S1. Raman data

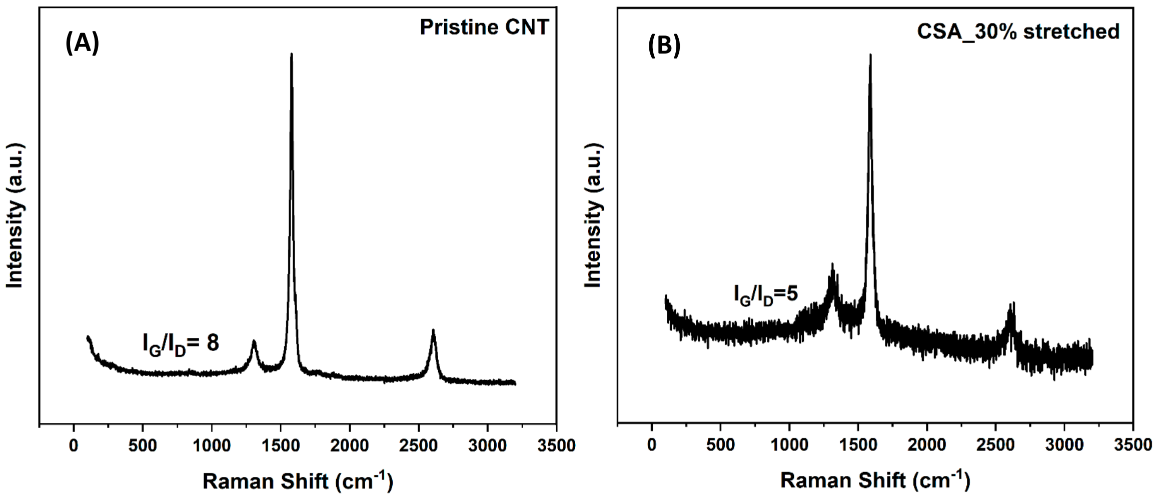

Figure S1. The Raman graph of as-synthesized CNT (A) and as-synthesized CNT after 30% CSA stretched (B).

S2. Mechanical properties measurement

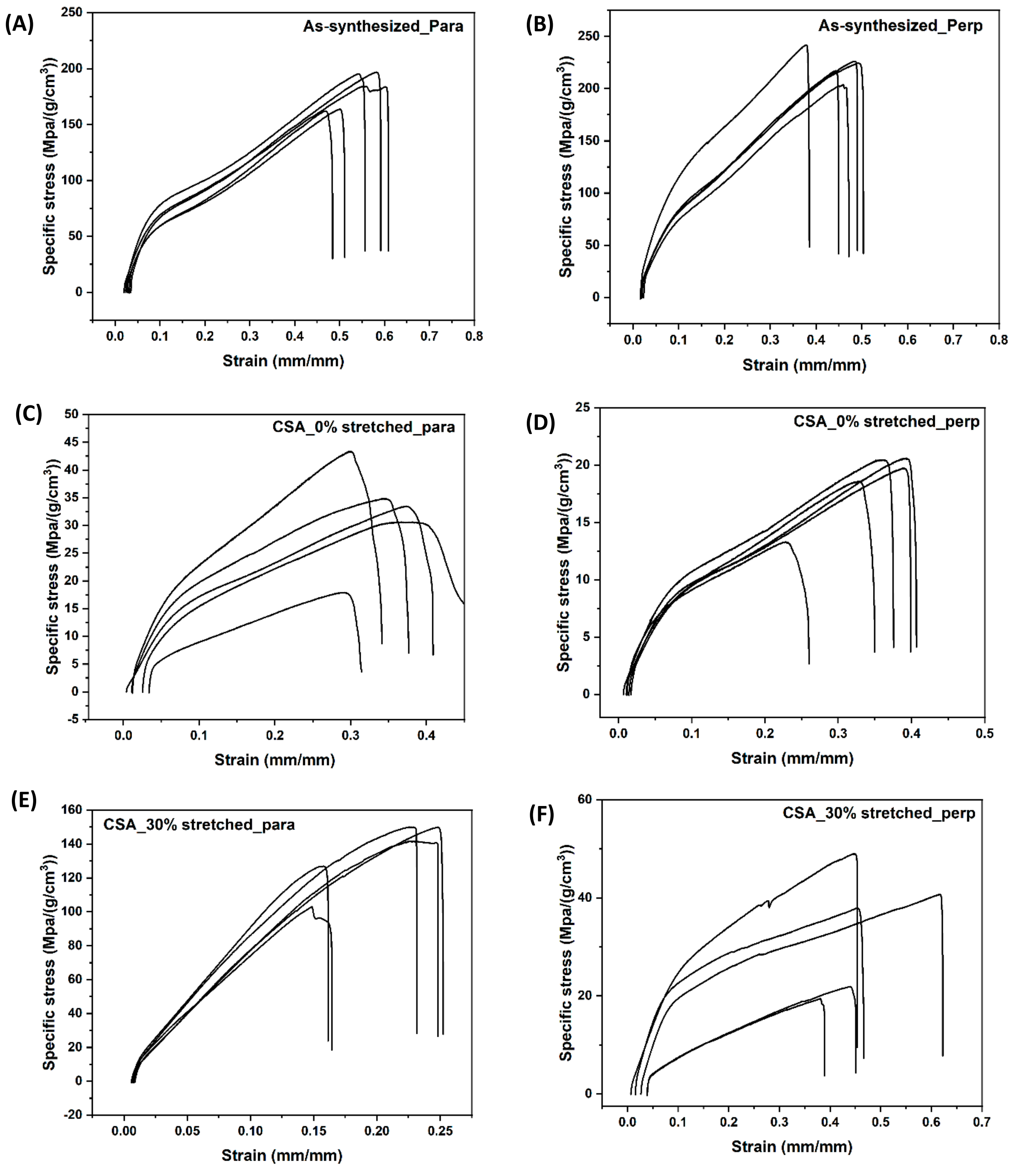

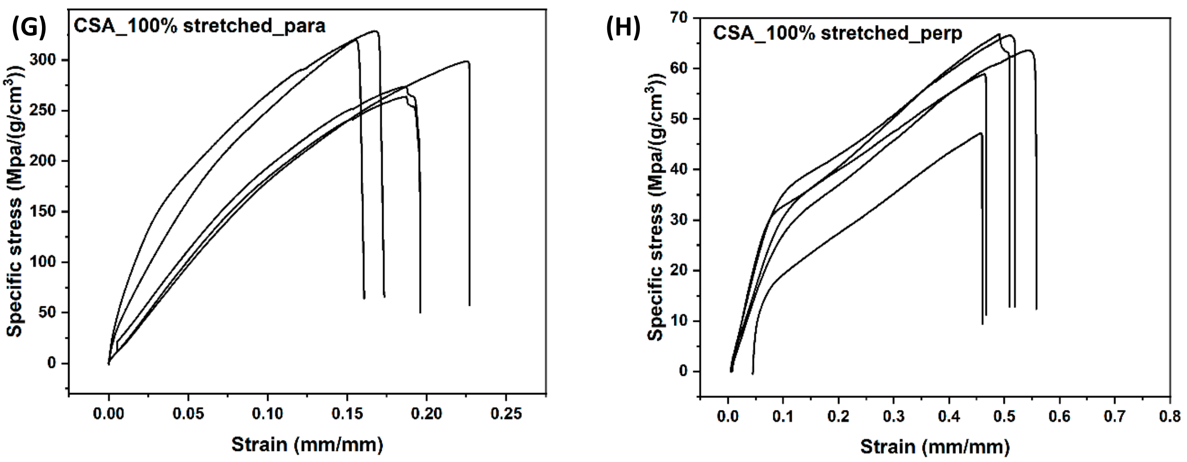

Figure S2. Tensile test of CNT samples after various treatments, the measurement of as-synthesized (without any treatment) CNT sample from parallel direction (A) and perpendicular direction (B); CNT sample after 0% stretched in the CSA solution from parallel direction (C) and perpendicular direction (D); CNT sample after 30% stretched in CSA solution from parallel direction (E) and perpendicular direction (F); CNT sample after 100% stretched in CSA solution from parallel direction (G) and perpendicular direction (H).

S3. Sheet conductance

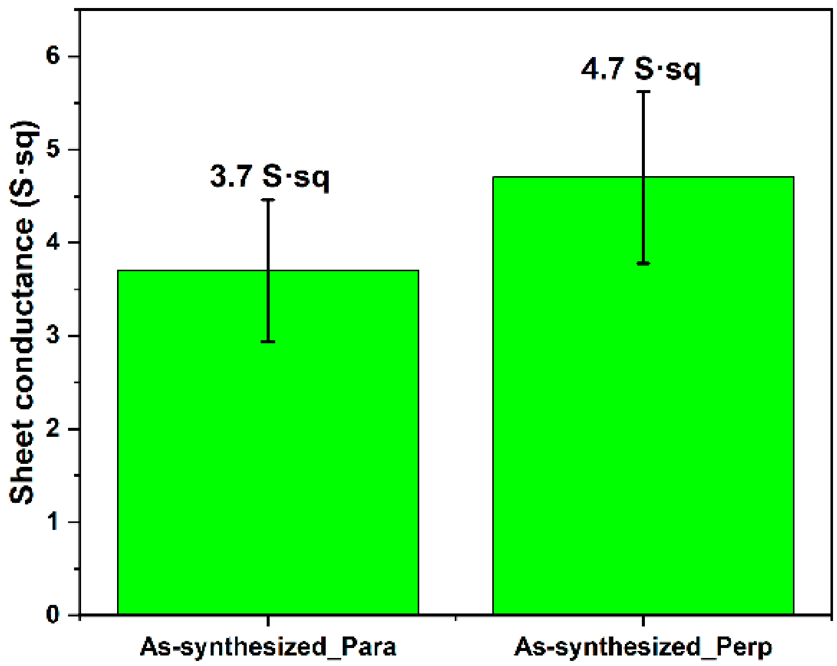

Figure S3. Sheet conductance of as-synthesized CNT sheet in parallel and perpendicular directions.

S4. Heating performance graph of various samples

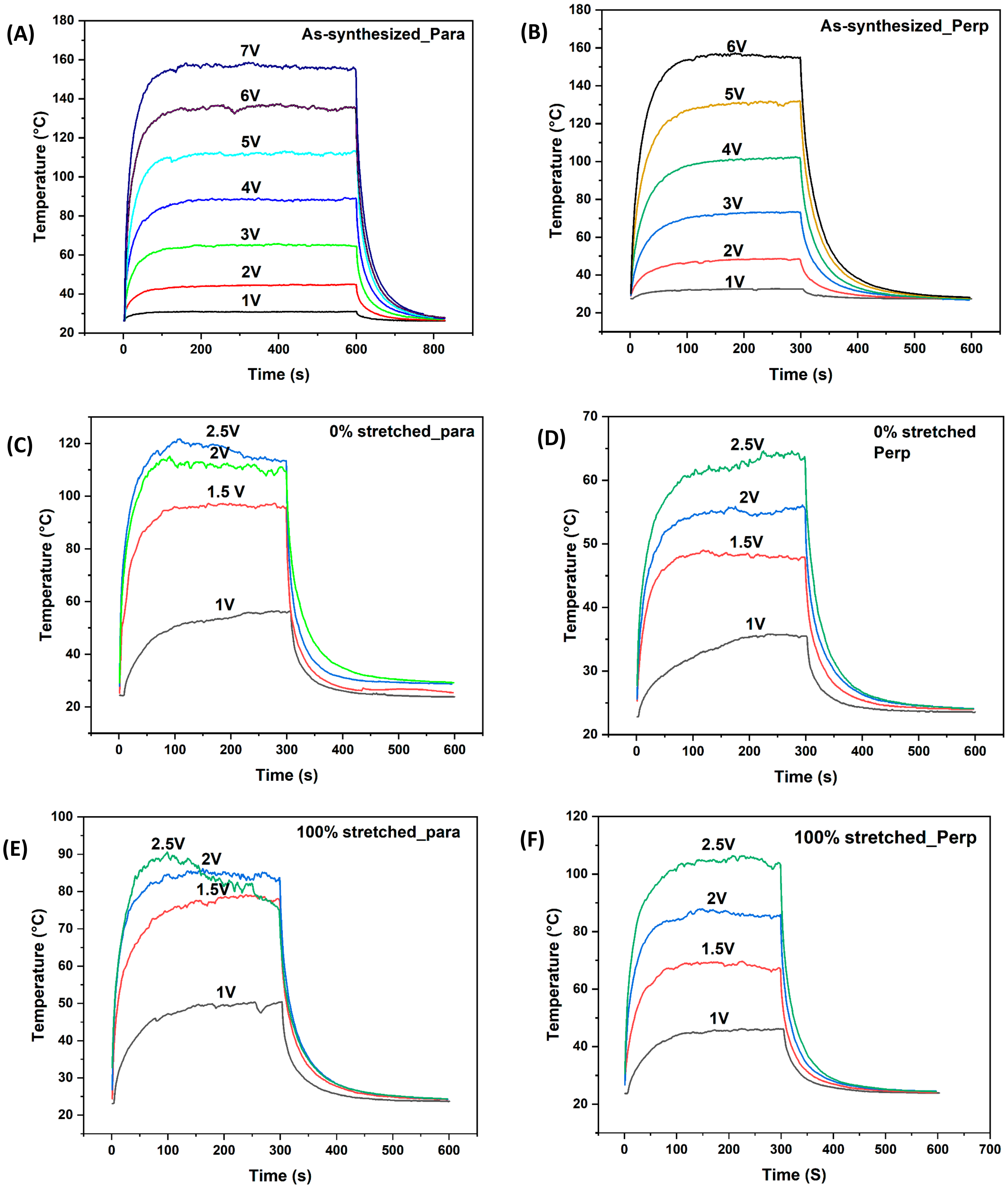

Figure S4. Heating performance under different voltages from samples: as-synthesized CNT in parallel direction (A) and perpendicular direction (B); 0% stretched in parallel direction (C) and perpendicular direction (D); 100% stretched in parallel direction (E) and perpendicular direction (F).

Table S1. Heating information of various stretched CNT sheets.

Sample	Current (A)	Voltage (V)	Electrical power input (W)	Maximum temperature (°C)	Time to reach maximum temperature (s)	Time to reach half of the maximum temperature (s)	Time to drop to half of the maximum temperature (s)	Temperature rise in the first 30s (s)
Pristine parallel	0.46	2.5	0.152	50.3	248	6	118	4.3
Pristine perpendicular	0.182	2.5	0.46	45.4	247	0	353	14.1
0% stretched parallel	0.693	2.5	1.39	121.5	105	5	206	78.7
0% stretched perpendicular	0.368	2.5	0.92	64.6	225	2	120	27.5
30% stretched parallel	0.9	2.5	2	183.6	83	<3	225	147.5
30% stretched perpendicular	0.774	2.5	1.95	115.1	91	4	234	71.6
100% stretched parallel	0.51	2.5	1.28	90.6	99	4	222	53.4
100% stretched perpendicular	0.614	2.5	1.54	106.2	210	5	114	60.3
